# Advising Consumption of Green Vegetables, Beef, and Full-Fat Dairy Products Has No Adverse Effects on the Lipid Profiles in Children

**DOI:** 10.3390/nu9050518

**Published:** 2017-05-19

**Authors:** Ellen José van der Gaag, Romy Wieffer, Judith van der Kraats

**Affiliations:** 1 Ziekenhuisgroep Twente, Hengelo, Geerdinksweg 141, Hengelo 7555 DL, The Netherlands; e.gaagvander@zgt.nl (E.J.v.d.G.); judithvanderkraats@hotmail.com (J.v.d.K.); 2 Isala Zwolle, Dokter van Heesweg 2, Zwolle 8025 AB, The Netherlands; romywieffer@gmail.com

**Keywords:** children, dietary advice, full-fat dairy products, green vegetables, beef, cholesterol, lipid profile, BMI, cardiovascular risk factors

## Abstract

In children, little is known about lipid profiles and the influence of dietary habits. In the past, we developed a dietary advice for optimizing the immune system, which comprised green vegetables, beef, whole milk, and full-fat butter. However, there are concerns about a possible negative influence of the full-fat dairy products of the diet on the lipid profile. We investigated the effect of the developed dietary advice on the lipid profile and BMI (body mass index)/BMI-*z*-score of children. In this retrospective cohort study, we included children aged 1–16 years, of whom a lipid profile was determined in the period between June 2011 and November 2013 in our hospital. Children who adhered to the dietary advice were assigned to the exposed group and the remaining children were assigned to the unexposed group. After following the dietary advice for at least three months, there was a statistically significant reduction in the cholesterol/HDL (high-density lipoproteins) ratio (*p* < 0.001) and non-HDL-cholesterol (*p* = 0.044) and a statistically significant increase in the HDL-cholesterol (*p* = 0.009) in the exposed group, while there was no difference in the BMI and BMI *z*-scores. The dietary advice has no adverse effect on the lipid profile, BMI, and BMI *z*-scores in children, but has a significant beneficial effect on the cholesterol/HDL ratio, non-HDL-cholesterol, and the HDL-cholesterol.

## 1. Introduction

Little is known about cholesterol and lipid profiles in children, except from children known to have familiar dyslipidemia. However, concerns about the cholesterol levels are troubling parents when doctors advise to give full-fat dairy products to their children. Are these concerns realistic or not? At this moment, adult recommendations are also used for children. 

There are circumstances when full-fat dairy products are investigated for their possible positive contribution to different health aspects in children. One aspect is the functioning of the immune system, which is partly dependent on the nutritional status. Nutrients, such as vitamins and minerals, play an important role in the strengthening of the immune system. As a consequence, an adequate nutritional status, and thereby a strong immune system, might prevent infections [1,2,3,4,5,6,7]. 

In a previous study, we compared the dietary intake of children with recurrent respiratory infection (without immunological disorders) and healthy children [8]. These children usually have respiratory complaints without an adequate explanation, like immunological deficiencies. The outcomes showed that the group of children with recurrent infections eats less beef, natural milk, and green vegetables compared to the healthy children. 

Following this study, a nutrient-rich diet has been developed as a possible intervention for recurrent infections using the NEVO (Nederlands Voedingsstoffenbestand) tables, a Dutch nutrient database containing information about the nutrients of each food [9]. There are more international databases containing macro and micronutrients. We choose this database because this database contains the most information about the regular food that is eaten and sold in The Netherlands. 

The diet is based on foods high in nutrients that could support the immune system, namely green vegetables, beef, whole milk, and butter (Table 1). This are also the food groups that are not frequently consumed by children with recurrent infections. Compared to other vegetables, green vegetables contain more zinc, vitamin A, and vitamin C. Beef contains more iron, zinc, vitamin A and vitamin E compared with other types of meat [9]. These nutrients have immune supporting effects and play a role in the antiviral mechanisms, which could positively affect recurrent upper respiratory tract infections [2,3,4,5,6,7]. Looking at the full-fat dairy products, whole milk, and butter are a source of lipids, vitamins, and essential fatty acids, such as linoleic acid and alpha-linolenic acid [9]. The lipids can act as a carrier for vitamins A, D, E, and K, [10] which can have a positive effect on the immune system [9,10]. In addition, the extra fats in whole milk have anti-microbial properties and can act as bacteriostatics [9,11].

This previous study showed that the dietary advice had significant positive effects on the length and gravity of respiratory tract infections in children [14]. Furthermore, another study showed that the same dietary advice decreases some symptoms of medically unresolved fatigue in children [1,15].

Strengthening the immune system just by changing food habits might be a solution for many patients with recurrent infections but without an immunological disorder or for patients with medically unresolved fatigue. However, there are thoughts that the saturated fats in the recommended whole milk and butter could have a negative influence on the lipid profile and/or the risk of cardiovascular disease. The National Heart Foundation of Australia states that the intake of saturated fatty acids is highly associated with an increased risk of coronary heart disease due to elevated LDL-cholesterol (low-density lipoproteins cholesterol) and serum cholesterol levels [16]. The American Heart Association (AHA) and American Academy of Pediatrics advise the use of dairy products that are fat-free or low in fat, in order to minimize the intake of saturated fat. They mention that a decline in saturated fat and cholesterol intake has been associated with a reduction in cardiovascular disease [17]. The Dutch Centre of Food recommends replacing saturated fats with unsaturated fats, which should lower the risk of cardiovascular disease [18]. 

Recently, conflicting findings have been reported regarding the association of saturated fats and the risk of cardiovascular disease. Several studies show no evidence for the assumed association and some even describe an inverse association [19,20,21].

The aim of our study was to determine whether the developed dietary advice—relatively high in saturated fats—has an influence on the BMI (body mass index) of children and on risk factors of cardiovascular disease. The total cholesterol/HDL (high-density lipoproteins) ratio is an important predictor of later risk of cardiovascular disease [22,23]. Additionally, the American Academy of Pediatrics recommends non-HDL concentration as an important benchmark for the screening of cardiovascular risk in children [24]. Therefore, we used the lipid profile of children in order to determine whether the dietary advice with its beneficial effect on at least respiratory tract infections in children can be safely used. 

## 2. Materials and Methods 

The present study is a non-randomized retrospective cohort study. The determination of the lipid profile of the children was executed by blinded laboratory workers. The measurements of weight and height were not blindly executed. 

**Figure 1:**
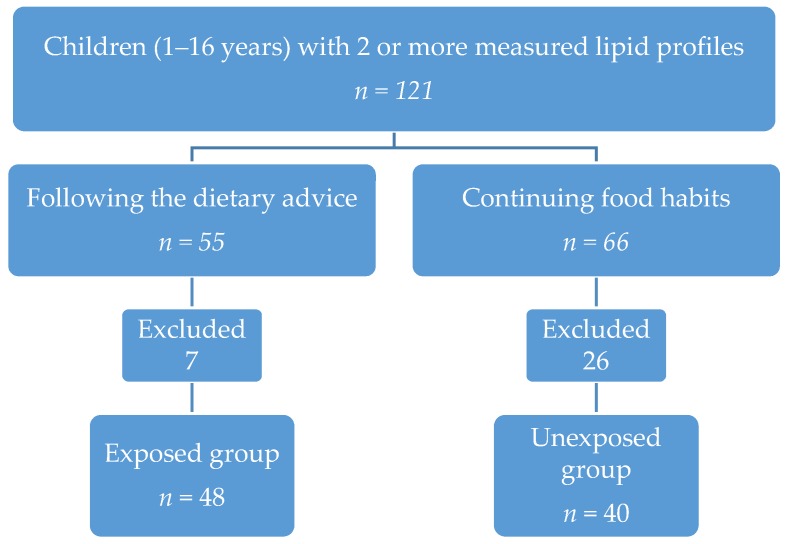
Schematic overview of the data collection.

We performed a laboratory search in our laboratory database for patient blood samples. Included in the search were children aged from 1 to 16 years with at least two measurements of a lipid profile in the period between June 2011 and November 2013 at hospital ZGT (Hospital Group Twente) Hengelo/Almelo in the Netherlands. Patient charts were hand-searched for dietary habits/advice. If no details were given in the patient charts, dietary habits were addressed as unknown. When no abnormalities were noted, we assumed it was according to the Dutch dietary guidelines [12]. Children who had followed the dietary advice were assigned to the exposed group and the remaining children were assigned to the unexposed group. A schematic overview of the data collection is shown in Figure 1. 

We excluded all children with a disorder that might influence the lipid profile, such as familiar hypercholesterolemia, hypothyroidism, diabetes mellitus type I and II, obesity, metabolic disorders, and medication which influences the lipid profile (according to [25]). As shown in Table 2, in the exposed group six patients were excluded based on the exclusion criteria described above, and one patient withdrew informed consent. Following the exclusion criteria, 26 patients were excluded in the unexposed group. 

The children visited the pediatric outpatient clinic for several complaints. In the exposed group, most of them suffered from recurrent infections, subclinical hypothyroidism or tiredness. The unexposed group consisted of children with recurrent infections, abdominal complaints, epilepsy, failure to thrive, behavioral disorders. 

The dietary advice, based on the NEVO tables [9], consists of eating beef three times a week, green vegetables five times a week (both age-related portions, according to the Dutch Center of Food), at least one glass (200 mL) of full-fat milk (3.4% fat) each day, and the use of five grams per slice of bread of natural butter (80% fat) for at least three months. Each item of the advice counted for 25% and children had to score at least 75% to meet the criteria of the exposed group. All other dietary habits remained unchanged. The children who did not follow the dietary advice were included in the unexposed group. For ethical reasons we were not allowed to approach them and had to assume that there were no large changes in their food habits during the period of follow-up. 

We recorded information of all children from both groups: gender, age, weight, height, duration, and degree of following the dietary advice, lipid profile at the time of presentation, and follow-up. 

The height of the children was measured with a vertical ruler. The children were weighed in underwear and all measurements were performed by a pediatrician. The children’s BMI was calculated by dividing their weight in kilograms by the square of their height in meters. The BMI *z*-score is calculated on the basis of gender, age, height, and weight [26]. The BMI *z*-score can be calculated only from the age of 24 months. This means that no BMI *z*-score was calculated in children younger than two years. These data were calculated, but not added in the tables, due to lacking data in the younger children.

Both for the start of the dietary advice, and at the end of the follow-up, the lipid profile was determined in all children. At the time of blood collection by venapuncture the children had an empty stomach, as nutrition can affect LDL and triglyceride concentrations [27]. The lipids from the lipid profile are total cholesterol, high-density lipoprotein cholesterol (HDL-C), cholesterol/HDL ratio, low-density lipoprotein cholesterol (LDL-C), triglycerides (TG), and non-HDL. The lipid profile was measured by enzymatic colorimetric techniques with the COBAS 6000 (Roche Diagnostics, Almere, The Netherlands). The LDL was calculated with Friedewald’s formula: LDL = total cholesterol − HDL − (0.45 × TG). The primary outcome of this study, the cholesterol/HDL ratio, was calculated by dividing the total cholesterol by HDL cholesterol [23]. The non-HDL can be calculated by the following formula: total cholesterol − HDL cholesterol = non-HDL cholesterol (non-HDL). 

We used SPSS Statistics 20 (SPSS Inc., Chicago, IL, USA) to execute our data analysis. Normality was checked by visual expectation of histograms and Shapiro-Wilk test. Continuous variables were expressed as the mean with the standard deviation (SD) or the median with the interquartile range (IQR); categorical variables were expressed as counts with corresponding percentages. Differences in baseline characteristics between groups was tested using an independent *t*-test or Mann-Whitney (continuous variables) or Pearson’s chi-square (categorical). To test changes of the lipid profile between measurements within each group a paired *T*-test or Wilcoxon was used. Concerning the BMI and BMI *z*-score, several data were lacking. Therefore, the BMI and BMI *z*-scores were tested using mixed models analysis. For all comparisons, a *p*-value ≤ 0.05 was regarded as significant. 

## 3. Results

### 3.1. Baseline Data

The baseline data of the unexposed and exposed group are presented in Table 3. The demographic characteristics, period of follow-up, the lipid profiles, and the BMI characteristics did not differ significantly at the start of this study. 

### 3.2. Changes within Groups

The baseline, follow up and differences in lipid profile within the two groups between the start and follow-up are shown in Table 4. In the exposed group, the HDL-cholesterol increased significantly with 0.14 mmol/L (*p* = 0.009), 95% CI (−0.24 to −0.04) (confidence interval). The cholesterol/HDL ratio was significantly reduced (*p* < 0.001), 95% CI (0.35–0.84), as was the non-HDL (*p* = 0.044), 95% CI (0.01–0.34). The decrease in the cholesterol/HDL is caused by the significant increase in the HDL-cholesterol. The total cholesterol did not change significantly and barely affects the cholesterol/HDL ratio. No significant changes occurred in the BMI and BMI *z*-score (a change of −0.06) in the exposed group. There were no significant changes of the lipid profile or BMI and BMI *z*-score (change of 0.09) in the unexposed group. 

## 4. Discussion

Our research shows that consumption of green vegetables, beef, whole milk, and butter has no adverse effect on the lipid profile in children. The dietary advice, no advice with respect to carbohydrate intake, but relatively high in saturated fats is even shown to have a favorable effect on the lipid profile: it gave a significant increase in HDL cholesterol, and a decrease in non-HDL cholesterol and the cholesterol/HDL ratio. 

In a previous study the dietary advice has been shown to have a significant improving effect on the incidence and duration of recurrent respiratory tract infections [15]. This nutritional advice will probably be discouraged by major national and international organizations since the idea exists that saturated fats have a negative effect on the lipid profile and/or the cholesterol/HDL ratio and, thus, increases the risk of cardiovascular disease. 

The American Heart Association and the American Academy of Pediatrics recommend not offering any whole-milk products to children, because of the higher concentrations of saturated fats and, therefore, the increased risk of later cardiovascular disease [17]. The Dutch Nutrition Centre recommends that children should not eat full-fat products at all, due to the relatively high concentration of saturated fats. According to the nutrition center the intake of saturated fats has a negative impact on the cholesterol/HDL ratio and, therefore, increases the risk of cardiovascular disease [18].

Over the years, various studies have been published discussing the relationship between saturated fatty acids and cardiovascular disease. The idea that consuming saturated fats can lead to death from cardiovascular disease has certainly not been confirmed by all studies. A meta-analysis of randomized trials showed that saturated fat has an increasing effect on HDL cholesterol. The increase in the HDL-cholesterol is greater when consuming saturated fats, compared to consuming unsaturated fats [28], which can contribute to a decrease in total cholesterol/HDL cholesterol ratio [29]. *The Lancet* published a systematic review of 61 prospective studies, which showed that higher HDL cholesterol levels reduce the risk of death from cardiovascular disease [30].

Contrary to expectations, a large meta-analysis by Siri-Tarino and colleagues shows that there is no significant link between the consumption of saturated fats and an increased risk of cardiovascular disease in general and coronary heart disease in particular [20]. In line with this, a meta-analysis by Skeaf and Miller commissioned by the World Health Organization concluded that the amount of saturated fats in a diet does not have an impact on the risk of coronary heart disease [31]. The American Heart Association claims that replacing saturated fat with carbohydrates lowers the risk of cardiovascular disease. In contrast, a meta-analysis of prospective studies shows that replacing saturated fat with carbohydrates leads to a significantly increased risk of cardiovascular disease [32]. This is supported by Musunuru, who concluded that it is not the saturated fats, but the carbohydrates in a diet that cause atherogenic dyslipidemia [33]. 

Next to the inconsistent data about dairy fats and cardiovascular risk factors, there are also inconsistent data about the risk of dairy fat on developing diabetes mellitus. A recent study from the Nurses’ Health Study and the Health Professionals Follow-Up Study show a protective effect of high plasma dairy fatty acid concentrations and lower incidence of diabetes mellitus [34].

As an alternative to butter with its saturated fats, margarine was developed. This “skinny” dairy product is enriched with “healthy” omega-6 fatty acids. However, the replacement of saturated fatty acids and trans-fatty acids by omega-6 fatty acids is associated with an increased risk of coronary heart disease and overall mortality [35]. We now know that omega-6 fatty acids have pro-inflammatory characteristics while omega-3 fatty acids have anti-inflammatory ones. A diet with a large amount of omega-6 fatty acids and a high omega-6/omega-3 ratio enhances the development of diseases such as cancer, cardiovascular disease, inflammatory and autoimmune diseases. In contrast, high levels of omega-3 fatty acids have suppressive effects on those diseases [36]. The investigated dietary advice contributes to a good fatty-acid balance due to its green vegetables, which contain a relatively high amount of omega-3 fatty acids and are low in omega-6 fatty acids [9]. Recently, a study showed that people who eat a lot of green leafy vegetables have a 32% lower risk of myocardial infarction [37]. In addition, green vegetables have other positive effects concerning health, such as reducing the risk of many forms of cancer [38,39]. Additionally, the dietary advice contributes to the inhibition of oxidation of LDL cholesterol, a crucial step in atherosclerosis, with its relatively high levels of Vitamin A and E in beef, compared to other types of meat [40]. 

The BMI and BMI *z*-scores in the exposed group did not significantly change during the months of follow-up. If we calculate the caloric intake of the dietary advice, using age-adequate quantities advised by the Dutch Food Center [12], the diet contains 94 more calories compared to a diet with identical quantities of low-fat milk and margarine [9]. By contrast, beef contains 1.5 times fewer calories compared to, for example, pork, which has 82 calories per serving [9,41]. This almost neutralizes the extra calories ingested by a child with the intake of whole milk and butter. Additionally, whole milk has a favorable glycemic control and, thereby, possibly an inhibitory effect on appetite and food intake [42]. Several investigations show that a higher intake of dairy products does not increase body weight, results that are consistent with the results of our study [43,44]. 

This study suggests that diet quality can have some benefits for children. However, one of the limitations of this study is the retrospective design. Adherence to the dietary advice was retrospectively controlled through evaluative questions during the consultation with the pediatrician. A more reliable way of checking the nutritional advice is to let patients fill out a daily food questionnaire.

Due to the retrospective design the food habits of the unexposed group could not all be traced. In this case we had to assume that they did not consume full fat dairy (in The Netherlands semi-skimmed milk and low-fat butter are advised) and no changes in diet occurred during follow-up. In a research design such as a randomized controlled trial, the unexposed group could also fill out a food questionnaire so that any changes in diet can be detected. 

Following the retrospective design of this study the unexposed and exposed group could not be randomized. A probable advantage is that the patients (and/or their parents) in the exposed group were possibly more motivated to follow the diet given the fact that they chose to follow the diet themselves. 

There were missing values in the BMI and, thereby, the BMI *z*-scores of the children, so that the conclusions of BMI and BMI *z*-score are based on a smaller number of patients than we included. Furthermore, the mean period of follow-up was 4.4 months, which means that we cannot draw conclusions about these outcomes in the long term. We require long-term follow-up studies to evaluate the course of the lipid profile.

## 5. Conclusions

This retrospective study shows diet quality in childhood can have some useful benefits. Earlier, it was shown that a dietary advice of green vegetables, beef, whole milk, and full-fat butter reduces the number of days with a respiratory tract infection in children. In this study we have shown that the dietary advice has no adverse effect on the lipid profile, BMI, and BMI *z*-score in children. Conversely, the dietary advice has a significant beneficial effect on the HDL-cholesterol, cholesterol/HDL ratio, and non-HDL-cholesterol. The dietary advice can, therefore, be safely recommended and might be beneficial for children with recurrent respiratory tract infections. However, the findings of this retrospective study should be further investigated in randomized controlled trials.

## Figures and Tables

**Table 1:** Nutrients in food products of the dietary advice compared to other food products (according to the NEVO tables [9]).

Food Product	Nutrients per 100 Grams									
	Vitamin A (ug)	Vitamin D (ug)	Vitamin E (mg)	Iron (mg)	Zinc (mg)	Calorie (kcal)	Saturated Fats (g)	Total Unsaturated fats (g)	N-3 Fats (g)	Linoleic Acid (N-6 Fat) (g)
Spinach cooked	652	-	3.5	2.4	1.20	25	0.1	0.7	0.5	0.1
Broccoli cooked	116	-	2.5	0.9	0.62	27	0.1	0.2	0.1	-
Cauliflower cooked	0	-	0.1	0.3	0.26	23	0.1	0.2	0.2	-
Chicory cooked	1	-	0.2	0.2	0.17	17	-	0.1	-	0.1
Beef > 10% fat	68	0.5	2.4	2.8	5.84	277	6.2	10.5	0.2	2.9
Chicken breast	18	0.1	1.1	0.7	0.74	158	1.4	1.8	0.1	0.8
Pork 10%–19% fat	25	0.6	1.1	1.0	2.65	378	5.4	10.1	0.2	3.2
Butter	903	1.2	2.5	0.1	0.09	737	52.9	19.9	0.5	1.3
Margarine	800	7.5	9.5	0.1	-	349	8.5	34.5	5.9	19
Whole milk	36	-	0.1	-	0.46	62	2.2	0.8	-	0.1
Skimmed milk	1	-	-	-	0.46	35	0.1	-	-	-
Adequate intake or recommended dietary allowance/day for children [12,13]	♂/♀ 2–5 years: 350 ug	♂/♀ 4–8 years: 10 ug	♂/♀ 2–5 years: 5 mg	♂/♀ 2–5 years: 8 mg	♂/♀ 2–5 years: 6 mg	4–8 years: ♂1720 kcal ♀1552 kcal	♂/♀ 4–8 years: 10 En%	♂/♀ all ages: 8–38 En%	♂/♀ 4–8 years: 0.15–0.2 g	♂/♀ 4–8 years: 2 En%

**Table 2:** Overview of the excluded patients.

Exposed Group	(*n* = 55)	Unexposed Group	(*n* = 66)
Incomplete lipid profile	2	Incomplete lipid profile	5
Familiar hypercholesterolemia	2	Familiar hypercholesterolemia	3
Obesity	1	Obesity	13
Age < 1 year or > 16 years	1	Age < 1 year or > 16 years	1
Diabetes mellitus	0	Diabetes mellitus	3
Metabolic disorder	0	Metabolic disorder	1
Medication	0	Medication	0
Dropouts	1	Dropouts	0
Exposed group	(*n* = 48)	Unexposed group	(*n* = 40)

**Table 3:** Baseline characteristics of the unexposed and exposed group.

Characteristic	Unexposed Group *n* = 40	Exposed Group *n* = 48	*p*-Value
Gender (n, %)MenWomen	24 (60%) 16 (40%)	25 (52%) 23 (48%)	0.457
Age (years) (median, IQR)	4.7 (2.3–9.0)	2.6 (1.6–8.0)	0.102
Follow-up (months)(median, IQR)	5.0 (4.0–8.0)	4.5 (4.0–8.8)	0.744
BMI (median, IQR)	15.9 (15.1–17.5)	16.7 (15.4–18.5)	0.408

IQR (interquartile range); SD (standard deviation).

**Table 4:** Changes in lipid profile and BMI of both groups between the start and end of follow-up.

Measurements	Unexposed Group *n* = 40				Exposed Group *n* = 48			
	Baseline	Follow-up	Change (95%-CI/IQR))	*p*-Value	Baseline	Follow-up	Change (95%-CI)	*p*-Value
Total cholesterol (mmol/L) (median, IQR)	4.05 (3.83–4.70)	4.20 (3.70–4.68)	−0.06 ^a^ (−0.11–0.22)	0.581 ^d^	4.20 (3.5–5.0)	4.35 (3.7–4.7)	−0.03 ^a^ (−0.25–0.18)	0.738 ^c^
HDL-cholesterol (mmol/L) (median, IQR)	1.35 (0.93–1.59)	1.30 (0.95–1.57)	−0.01 ^b^ (−0.19–0.12)	0.842 ^c^	1.17 (0.88–1.48)	1.35 (1.12–1.53)	0.14 ^a^ (0.04–0.24)	0.009 ^d^
Cholesterol/HDL (mmol/L) (median, IQR)	3.45 (2.57–4.70)	3.40 (2.53–4.45)	0,00 ^b^ (−0.35–0.38)	0.883 ^d^	3.75 (3.00–4.95)	3.15 (2.80–4.95)	−0.30 ^b^ (−1.2–0.17)	< 0.001 ^c^
Triglycerides (mmol/L) (median, IQR)	0.96 (0.70–1.93)	1.00 (0.80–1.47)	0.05 ^b^ (−0.38–0.30)	0.821 ^d^	1.10 (0.80–1.67)	1.05 (0.80–1.50)	−0.07 ^a^ (−0.31–0.16)	0.469 ^d^
LDL-cholesterol (mmol/L) (median, IQR)	2.30 (2.00–2.80)	2.30 (1.90–2.88)	0.00 ^a^ (−0.15–0.13)	0.852 ^c^	2.55 (1.70–3.00)	2.40 (1.93–2.80)	−0.10 ^b^ (−0.60–0.30)	0.384 ^c^
Non-HDL cholesterol (mmol/L) (median, IQR)	3.01 (2.54–3.49)	2.83 (2.40–3.39)	−0.06 ^a^ (−0.21–0.08)	0.384 ^c^	3.14 (2.56–3.61)	2.98 (2.45–3.28)	−0.17 ^a^ (–0.34—0.01)	0.044 ^d^
BMI (median, IQR)	15.9 (15.1–17.5)	15.8 (15.1–17.5)	0.24 ^a^ (-0.05-0.54)	0.178 ^d^	16.7 (15.4–18.5)	16.0 (14.9–18.0)	0.00 ^b^ (−0.63–0.30)	0.719 ^d^

^a^ Normally distributed (mean, 95% CI); ^b^ non-normally distributed (median, IQR); ^c^ paired *t*-test; ^d^ Wilcoxon signed rank test.
